# Gene expression-based immune infiltration analyses of renal cancer and their associations with survival outcome

**DOI:** 10.1186/s12885-021-08244-2

**Published:** 2021-05-24

**Authors:** Lei Chen, Liang Yin, Zilong Qi, Jinmin Li, Xinning Wang, Kun Ma, Xiangyang Liu

**Affiliations:** Department of Pediatric Surgery, Cangzhou Central Hospital, No.16 Xinhua West Road, Cangzhou, 061000 Hebei China

**Keywords:** Renal cancer, Tumor-infiltrating immune cells, Proportion, Prognosis

## Abstract

**Background:**

Renal cancer is a common malignant tumor with an increasing incidence rate.

**Methods:**

In this study, based on the gene expression profiles, we analyzed the compositions of tumor-infiltrating immune cells (TIICs) in renal cancer and paracancerous samples using CIBERSORT. The proportions of 22 TIICs subsets in 122 paired renal carcinoma and paracancerous samples, and 224 Wilms tumor (WT) samples varied between intragroup and intergroup.

**Results:**

After analyzed the difference of TIICs composition between renal cancer and paired paracancerous samples, we found that M0 macrophages and CD8 T cells were significantly elevated, while naive B cells were significantly decreased in renal cancer samples compared with paracancerous samples. Survival analysis showed that high overall TIICs proportion*,* the low proportion of resting mast cells and the high proportion of activated memory CD4 T cells were associated with poor prognosis of renal cancer patients. In addition, 3 clusters were identified by hierarchical clustering analysis, and they presented a distinct prognosis. Cluster 1 had superior survival outcomes, while cluster 2 had an inferior survival outcome.

**Conclusions:**

Our study indicated that overall TIICs proportion, certain TIICs subset proportion, including resting mast cells and activated memory CD4 T cells, and distinct cluster patterns were associated with the prognosis of renal cancer, which was significant for the clinical surveillance and treatment of renal cancer.

**Supplementary Information:**

The online version contains supplementary material available at 10.1186/s12885-021-08244-2.

## Background

Renal cancer is a common malignant tumor with an increasing incidence rate over time [1, 2]. Wilms tumor (WT), also known as nephroblastoma, is the second most common solid abdominal organ tumor in children and the most frequent primary malignant renal cancer [3]. It accounts for 6% of pediatric cancers, and an estimated 95% of renal cancer is WT among children [4]. Clinically, WT or nephroblastoma is a common childhood tumor that is intimately linked to early kidney development and is often associated with persistent embryonic renal tissue and other kidney abnormalities, which is different from other types of renal cancer [5]. As an embryonic tumor of the kidney, WT arises from metanephric mesenchyme and presents high a incidence rate among 2–3-year-old children [6]. The treatment of WT is determined by several factors, such as genetic, pathologic, and demographic factors, and current therapies for WT consist of surgery, chemotherapy, and radiation therapy [3, 4]. Although the survival of WT has improved with the development of treatment strategies, the prognosis-related factors, including hematogenous and pulmonary metastasis, relatively high recurrence rate, and late effects, make the prospects of treatment noteworthy [7, 8]. Therefore, further investigation of the biological processes and underlying molecular mechanisms of WT may facilitate the prognosis improvement of patients.

Currently, increasing evidence has proved that the occurrence and development of tumors are associated with tumor cells as well as tumor microenvironment [9]. The tumor microenvironment is composed of complex components, such as mesenchymal stem cells, fibroblasts, and immune cells [10]. Among them, tumor-infiltrating immune cells (TIICs) are the pivotal components and are considered the leading players of the tumor microenvironment [11]. Accumulating studies have shown that TIICs are closely associated with the development and survival outcome of cancers. For example, infiltrated macrophages were found to promote the progression of prostate cancer, and NK cells showed anti-tumor effects against tumor development [12]. In colorectal cancer, TIICs were associated with patients’ clinical outcomes and considered key signatures for prognosis [13]. The immunohistologic features and immunotype of TIICs played a crucial role in metastatic melanoma and were related to the survival outcome [14]. In addition, dysfunctional infiltrating lymphocytes were observed in renal cell carcinoma (RCC), immunogenic renal cancer [15]. It was indicated that tumors might exert an impairment effect on the immune system, and more studies on the associations between TIICs and tumor occurrence and prognosis should be helpful for the application of immunotherapeutic strategies on renal cancer.

Immunohistochemistry is a commonly used method to evaluate the TIICs in tumors. However, the results are often inaccurate due to the extensive-expression of markers in non-immune cells [9]. In our research, the CIBERSORT algorithm was adopted to assess the TIICs subsets in renal cancer based on gene expression profiles retrieved from The Cancer Genome Atlas (TCGA). Meanwhile, we also analyzed the relationship of TIICs fraction and immune patterns with the survival outcome of renal cancer to explore the prognostic values of TIICs fraction and immune clusters in renal cancer.

## Methods

### Data source

We downloaded the gene expression profiles of renal cancer from the Gene Expression Omnibus (GEO, https://www.ncbi.nlm.nih.gov/geo/, Affymetrix HG-U133A platform) and The Cancer Genome Atlas (TCGA, www.cancergenome.nih.gov, Illumina HiSeq platform). The TCGA dataset was composed of 889 renal cancer samples and 128 paracancerous samples, including chromophobe renal cell carcinoma, kidney renal clear cell carcinoma, and kidney renal papillary cell carcinoma samples. The GEO dataset (access no. GSE31403) included 224 Wilms tumor (WT) samples.

### Tumor-infiltrating immune cells calculation

CIBERSORT (http://cibersort.stanford.edu), a deconvolution algorithm based on gene expression profiles [16], was used to calculate the relative proportions of 22 tumor-infiltrating immune cells (TIICs) subsets. CIBERSORT is able to evaluate the composition of TIICs with 547 barcode gene expression values, using *P*-value as a parameter for measurement of the confidence in results.

In addition, In the complex cancer immune microenvironment, cytotoxic T cells (Tc) and NK cells are two main effector cell types that can attack tumor cells directly. Upon exposure to transformed cells, cytotoxic T cells and NK cells secrete granzymes (a family of serine proteases) and perforin (a pore-forming protein) that will ultimately lead to target cell death. Thus, the local immune cytolytic activity can be quantified based on the transcript levels of perforin (PRF1) and granzyme A (GZMA). The first cytotoxin polymerizes and creates a channel in the membrane of the target cell. Through these pores, granzymes will then enter the cytoplasm and trigger a caspase cascade, composed of cysteine proteases that will ultimately lead to apoptosis [17]. GZMA is a tryptase that induces caspase-independent programmed cell death, and PRF1 serves as a pore-forming enzyme that regulates entry of granzymes into target cells [18]. Accordingly, it has been well-identified that the mean expression levels of genes GZMA and PRF1 represent the immune cytolytic activity of immune cells, which also reflect the fraction of TIICs [19–24]. Therefore, the expression levels of GZMA and PRF1 were determined to comprehensively assess the immune cytolytic activity and evaluate TIICs composition.

### Survival analysis

All 1241 samples were grouped according to results of CIBERSORT with *P*-values greater than or equal to 0.05 and less than 0.05, then the proportion of samples in each group and the average expression levels of genes GZMA and PRF1 were calculated. Seven samples of patiens without survival information were excluded, then based on kaplan-Meier method, survival analyses of the 1010 samples from TCGA, which were also stratified by *P*-value of 0.05 were performed by *survival* package (https://cran.r-project.org/web/packages/survival/) and *survminer* package (https://cran.r-project.org/web/packages/survminer/). With the relative proportions of 22 TIICs subsets as continuous variables, we calculated the hazard ratios (HR) using Cox regression analysis. We performed survival analyses to explore the effects of significant factors on survival outcomes.

### Clustering analysis

Based on K-mean method, cluster the samples with the relative content of immune infiltrated cells by R software v3.5.2.

### Statistical analyses

We used R language to calculate the proportions of 22 TIICs subpopulations in the 1241 samples from TCGA and GEO datasets. The correlation of the results from different datasets was analyzed by Pearson correlation analysis. The compositions of TIICs subpopulations in 122 paired cancer and paracancerous samples from TCGA were computed, and the difference in compositions was further analyzed. Besides, the fraction of TIICs subsets in WT samples from GEO was also calculated.

Then we stratified the 1241 samples by *P*-value of 0.05, and calculated the compositions of TIICs subsets as well as mean expression values of GZMA and PRF1 in the groups with *P* ≥ 0.05 and *P* < 0.05 respectively.

## Results

### Performance of CIBERSORT for TIICs evaluation in renal cancer

**Fig. 1:**
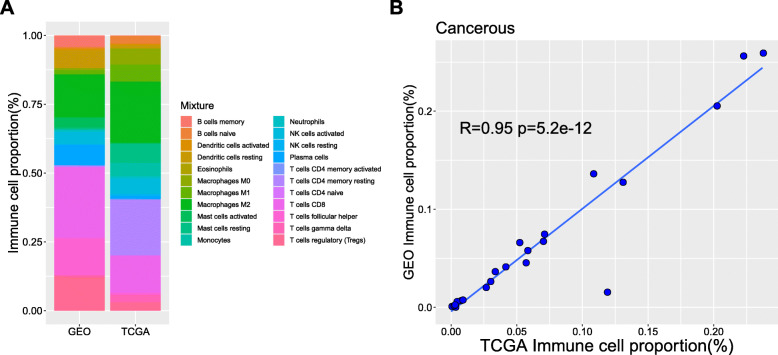
Renal cancer samples from GEO and TCGA had highly consistent immune cell proportion. **a** TIICs compositions of renal cancer and paracancerous samples from TCGA and GEO analyzed by CIBERSORT. **b** Proportions of TIICs subsets in renal cancer samples from TCGA and GEO

The TIICs composition was analyzed by CIBERSORT. As shown in Fig. 1a, the samples from GEO and TCGA presented distinct proportions of 22 TIICs subsets. As the renal cancer samples in GEO were all WT patients, we speculated that the TIICs in WT samples were significantly different from those in other renal cancer samples. Then we analyzed the correlation of immune cell proportions in renal cancer samples from TCGA and GEO. It was revealed that the renal cancer samples from the two datasets had highly consistent immune cell proportions (Fig. 1b), illustrating that CIBERSORT could evaluate the fraction of TIICs independently of data sources and platforms.

### Landscape of TIICs in renal cancer

**Fig. 2:**
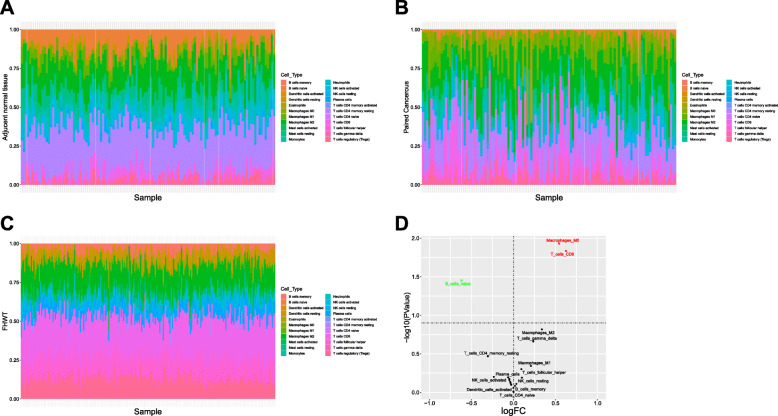
Obvious intragroup and intergroup differences in TIICs fraction were observed among paired renal cancer and paracancerous samples from TCGA, and WT samples from GEO. **a** Immune infiltration in paired paracancerous samples. **b** Immune infiltration in paired renal cancer samples. **c** Immune infiltration in WT samples. **d** Volcano plot of TIICs subsets proportions between paired renal cancer and paracancerous samples. Compared with paired paracancerous samples, M0 macrophages and CD8 T cells were significantly elevated, while naive B cells were significantly decreased in renal cancer samples

The compositions of TIICs subsets in 122 paired paracancerous and renal cancer samples from the TCGA and 224 WT samples from GEO were calculated by CIBERSORT, respectively. As shown in Fig. 2a-c and Table S1 the intragroup and intergroup differences in TIICs fractions were manifest. Thus, we inferred that the proportion of TIICs subsets was an inherent characteristic which varied significantly among different individuals. In addition, we analyzed the difference in TIICs composition between renal cancer and paired paracancerous samples. As shown in Fig. 2d, M0 macrophages and CD8 T cells were significantly elevated, while naive B cells were significantly decreased in renal cancer samples compared with those in paired paracancerous samples, and the results were basically consistent with previous researches [25, 26]. Moreover, the data in TCGA was performed by CIBERSORT (Fig. S1), the results were consistent with previous research [27].

### *P*-value of CIBERSORT represents the overall proportion of TIICs

**Fig. 3:**
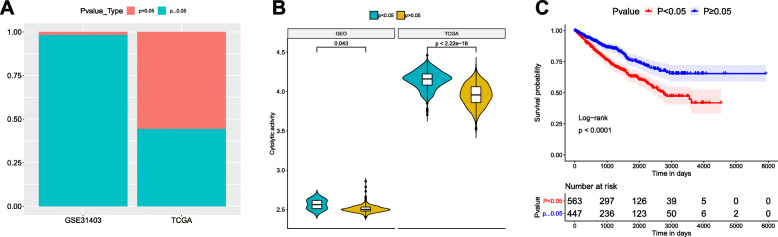
*P*-value of CIBERSORT represented the overall proportion of TIICs. **a** Proportions of samples with *P*-value < 0.05 and *P*-value ≥0.05 in GEO and TCGA datasets. **b** Immune cytolytic activity of samples with *P*-value < 0.05 and *P*-value ≥0.05 in TCGA and GEO cohort. **c** Survival curves of samples stratified by *P*-value of 0.05. Compared with renal cancer patients with *P*-value ≥0.05, patients with *P*-value < 0.05 had poor survival outcome (*p* < 0.0001)

It should be noted that instead of determining the actual values, CIBERSORT only calculates the relative ratios of TIICs subsets, which contributes to the dependency of results on each other. Therefore, we further analyzed the association between the *P*-value provided by CIBERSORT and TIICs composition. CIBERSORT *P*-value < 0.05 correlate with higher immune cell infiltrates, while no significantly different of the *P*-value ≥0.05 [28, 29]. Figure 3a showed that the proportions of samples with *P*-value < 0.05 and *P*-value ≥0.05 in TCGA and GEO were obviously different.

It has been well proved that the mean expression values of GZMA and PRF1 represent the immune cytolytic activity and are positively associated with TIICs proportions [19–24]. Accordingly, we futher evaluated the mean expression values, which represented the immune cytolytic activity in the system. After analyzing their mean expression values, we found that the samples with *P*-value < 0.05 had higher immune cytolytic activity in both TCGA and GEO cohorts (*p* < 2.22e-16 and *p* = 0.043, respectively, Fig. 3b and c). These results indicated that the proportion of TIICs in samples with *P*-value < 0.05 was higher in comparison to samples with *P*-value ≥0.05.

### Overall TIICs proportion, resting mast cells and activated memory CD4 T cells are associated with prognosis of renal cancer

To investigate the effects of overall TIICs proportion or 22 individual TIICs subset on renal cancer prognosis, the survival analyses of 1010 renal cancer samples with survival information were performed. The survival curve of renal cancer samples stratified by *P*-value of 0.05 showed that samples with *P*-value < 0.05 presented inferior survival outcome in comparison to samples with *P*-value ≥0.05 (*p* < 0.0001, Fig. 3d). It was suggested that high TIICs proportions might be associated with poor prognosis of renal cancer patients.

**Fig. 4:**
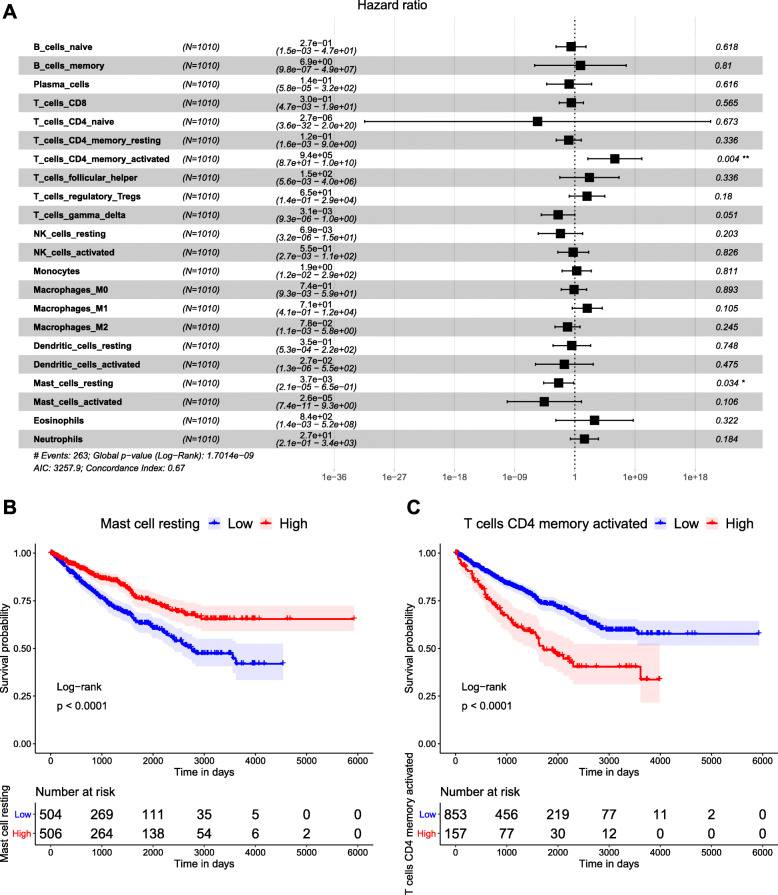
Decreased resting mast cells and elevated activated memory CD4 T cells were associated with poor prognosis of renal cancer patients. **a** Association of 22 TIICs subsets with survival outcomes of renal cancer patients. * and ** indicated statistical significance. **b** Survival curve of resting mast cells stratified by median proportion. **c** Survival curve of activated memory CD4 T cells stratified by median proportion

Subsequently, to further explore the effects of 22 individual TIICs subset on renal cancer prognosis, the univariate Cox regression analysis was conducted with 22 TIICs subsets as continuous variables. The relevant 95% confidence intervals and Hazard Ratios (HRs) were shown in Fig. 4a. It was found that activated memory CD4 T cells and resting mast cells were significantly associated with the prognosis of renal cancer patients (HR = 9.4e + 05, *p* = 0.004 and HR = 3.7e – 03, *p* = 0.034, respectively). The survival curves showed a low proportion of resting mast cells and a high proportion of activated memory CD4 T cells were related to poor prognosis of renal cancer patients (both *p* < 0.0001).

### Different immune clusters are associated with the prognosis of renal cancer

**Fig. 5:**
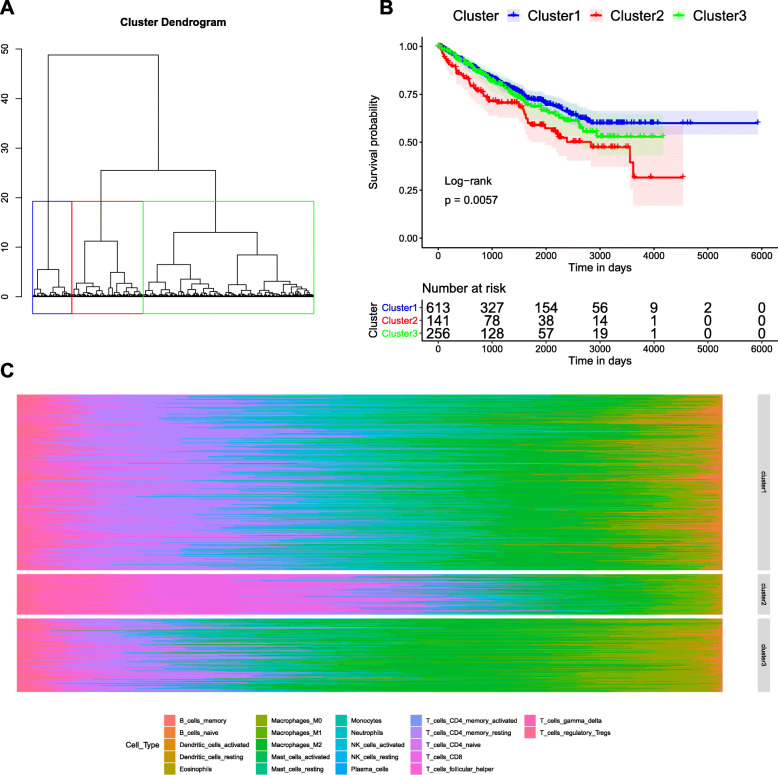
Immune clusters were associated with the prognosis of renal cancer patients. **a** Euclidean distance model identified three different immune clusters (cluster 1, cluster 2 and cluster 3). **b** Survival curves of cluster 1, 2 and 3. Cluster 1 presented superior survival outcomes, while cluster 2 presented inferior survival outcomes (*p* = 0.0057). **c** The 3 clusters exhibited different compositions of 22 TIICs subsets

The above results indicated TIICs alteration might affect the prognosis of renal cancer patients. Therefore, we speculated whether different immune clusters could be identified with the TIICs data. Firstly, the optimal number of clusters was determined as 3 using the within cluster sum of square errors (WSS) method (Fig. S1). Subsequently, the hierarchical clustering of the samples was conducted by the Euclidean distance model (Fig. 5a). After analyzed the association of different immune patterns with prognosis, cluster 1 exhibited superior survival outcomes, while cluster 2 exhibited inferior survival outcomes (*p* = 0.0057, Fig. 5b). Moreover, the compositions of 22 TIICs subsets is significantly different among the cluster 1, cluster 2, and cluster 3 (Fig. 5c). In additional, the immunes cell type abundances differ between clusters was revealed in violin plot Fig. S2 and clinical differences of the three clusters was showed in the Fig. S3. The results suggested that the relative proportion of 20 types of immune cells was significantly different in 3 clusters (*P* < 0.05, ANOVA). In Cluster1, with the best prognosis, the relative proportion of these 4 types of immune cells, including T cells CD4 memory resting, Macrophages M1, Mast cells resting and Monocytes, and Monocyte, were significantly higher than that of the other two clusters.

## Discussion

The tumor microenvironment is considered as a complex “society”, and exerts a regulatory effect on tumor progression with the participation of multiple cell types and extracellular matrix [30]. The various cell types in tumor microenvironment include mesenchymal stem cells, fibroblasts, endothelial cells, and immune cells [31]. It is believed that the TIICs are indispensable members of the tumor microenvironment and reflect the host immune reaction to tumors [11]. Accumulating evidence have proved that TIICs are associated with tumor invasion, metastasis, prognosis, and response to therapy [32, 33]. Given their important role, comprehensive researches on the TIICs in the tumor microenvironment may provide novel therapeutic approaches for tumors.

Numerous efforts have been devoted to exploring the specific role of TIICs in renal cancer by multiple approaches. Jensen HK et al. evaluated the TIICs in localized renal cell carcinoma by immunohistochemistry and found that neutrophils were independent prognostic signatures of survival outcome for localized renal cell carcinoma [34]. Donskov F et al. identified the positive correlation of CD57+ NK cells and negative correlation of neutrophils with the prognosis of metastatic renal cell carcinoma by immunohistochemistry [35]. Webster WS et al. proved that the infiltrated mononuclear cells were able to predict the survival outcome of renal cell carcinoma patients independently through hematoxylin and eosin staining and flow cytometric analysis [36]. Unlike the above researches, the TIICs in renal cancer were analyzed using a distinct approach in our study. We assessed the 22 TIICs subpopulations composition of renal cancer and paracancerous samples from TCGA and GEO using CIBERSORT algorithm based on their gene expression profiles. The TIICs subsets proportions of renal cancer samples from TCGA and GEO showed consistent results after correlation analysis. It was suggested that CIBERSORT was able to assess the composition of the TIICs subset, which was independent of data platforms and sources. Besides, CIBERSORT could be performed to characterize cell heterogeneity using RNA mixtures from nearly any source [37]. Many researchers used CIBERSORT for estimating the infiltration of immune cells, and there is no differences between using CIBERSORT on microarray datasets [38–40] and RNA sequencing datasets [41–43], even on both microarray datasets and RNA sequencing datasets [44], and chip platform [45]. Meanwhile, it has been reported that CIBERSORT has been used in several previous studies to analyzed the TIICs in the renal cell carcinoma, which have identified that CD8+ T cells were associated with prolonged overall survival and the potential biomarker relaed to CD8+ T cells, respectively [46, 47]. However, several researches reported that elevated CD8+ T cells were negatively related to prognosis in the patients with glioma [26] and hepatocellular carcinoma [25]. Besides, a different method of ESTIMATE algorithm has also been applied to analyze prognostic microenvironment-related genes and stromal and immune scores in Clear Cell Renal Cell Carcinoma [48]. In this study, our CIBERSORT analysis identified the landscape of TIICs in renal cancer and demonstated high TIICs proportions might be associated with poor prognosis of renal cancer patients. These data providing new evidence of the application of CIBERSORT in renal cancer and provide valuable information of TIICs in renal cancer patients.

Then we investigated the association of 22 individual TIICs subpopulation with the survival outcome of renal cancer patients by Cox regression analysis and survival analysis. The results showed that elevated proportion of activated memory CD4 T cells and decreased proportion of resting mast cells were associated with poor prognosis of renal cancer. It was known that the activated memory CD4 T cells could secret interleukin (IL) 17, a proinflammatory cytokine that promotes the proliferation and growth of cervical cancer [49]. It has been reported that IL-17 is able to promote the development of colorectal cancer and associate with poor prognosis [50]. In the present study, a high proportion of activated memory CD4 T cells were related to poor prognosis of renal cancer patients. Hence, we speculated that the increased proportion of activated memory CD4 T cells contributed to inferior survival outcomes in renal cancer through enhanced secretion of IL-17, which might be one of the possible mechanisms and needed to be validated further in the future investigations. Mast cells, which reside in vascularized tissues, have two states, including resting condition and activated condition [51]. They are involved in multiple tumor-related processes. In thyroid cancer, thyroid cancer cells activated the mast cells, which could secrete extensive proinflammatory, angiogenic, and growth factors and exert protumorigenic effects [51]. We inferred that the decreased proportion of resting mast cells, which reflected the enhancement of activated mast cells activities, was associated with a poor prognosis due to the protumorigenic effects of activated mast cells. In addition, a previous study showed a four immune-related genes signature based on CXCL2, SEMA3G, PDGFD, and UCN is closely associated with the prognosis of renal clear cell carcinoma [52]. In this study, we identified that TIICs proportion is closely correlated with the prognosis of renal cancer, which was diffent from the previous study.

In addition, a total of 3 immune clusters was identified by hierarchical clustering analysis. After analyzing their fractions, we found that the proportions of TIICs subpopulations presented remarkable difference in the different clusters. The survival analysis revealed that the survival outcomes were significantly diffenrt among these 3 clusters, in which cluster 1 exhibited superior survival outcomes, while cluster 2 exhibited inferior survival outcomes. These findings indicated that immune infiltrate is heterogeneously different in the renal cancer patients and the difference of immune infiltrate is closely associated with the prognosis of of renal cancer patients.

## Conclusions

In summary, the analysis of 22 TIICs proportions in renal cancer samples showed elevated activated memory CD4 T cells proportion, and decreased resting mast cells proportion predicted poor prognosis in renal cancer. Different immune clusters also presented distinct survival outcomes. The results might unveil novel prognosis prediction and immunotherapeutic strategies on renal cancer.

## Supplementary Information

**Additional file 1 Table S1.** CIBERSORT result.

**Additional file 2 Fig. S1**. Relative proportions of immune cells in TCGA dataset analyzed by CIBERSORT. **a** The colum plot of 22 types of immune cells in TCGA renal cancer and paracancerous samples. **b** The box plot of 6 types of immune cells with significant differences between renal cancer and paracancerous samples.

**Additional file 3 Fig. S2**. Selection of the optimal number of clusters.

**Additional file 4 Fig. S3**. The violin plot of immune cell type abundances differ between clusters. Violin colors represent different clusters, and the vertical axis represents the relative proportion of immune cells.

**Additional file 5 Fig. S4**. The violin plot of clinical characters in three clusters. Violin colors represent different clusters, and the vertical axis represents samples. **a** Age. **b** Sex. **c** Stage.

## Data Availability

The datasets analysed during the current study are available in the TCGA and GEO repositories, www.cancergenome.nih.gov and https://www.ncbi.nlm.nih.gov/geo/ (access no. GSE31403).
