# Supplementary figures and images for: Gene expression-based immune infiltration analyses of renal cancer and their associations with survival outcome
Source: BMC Cancer. 2021 May 24;21:595. doi: 10.1186/s12885-021-08244-2 (PMC8146654; doi:10.1186/s12885-021-08244-2)

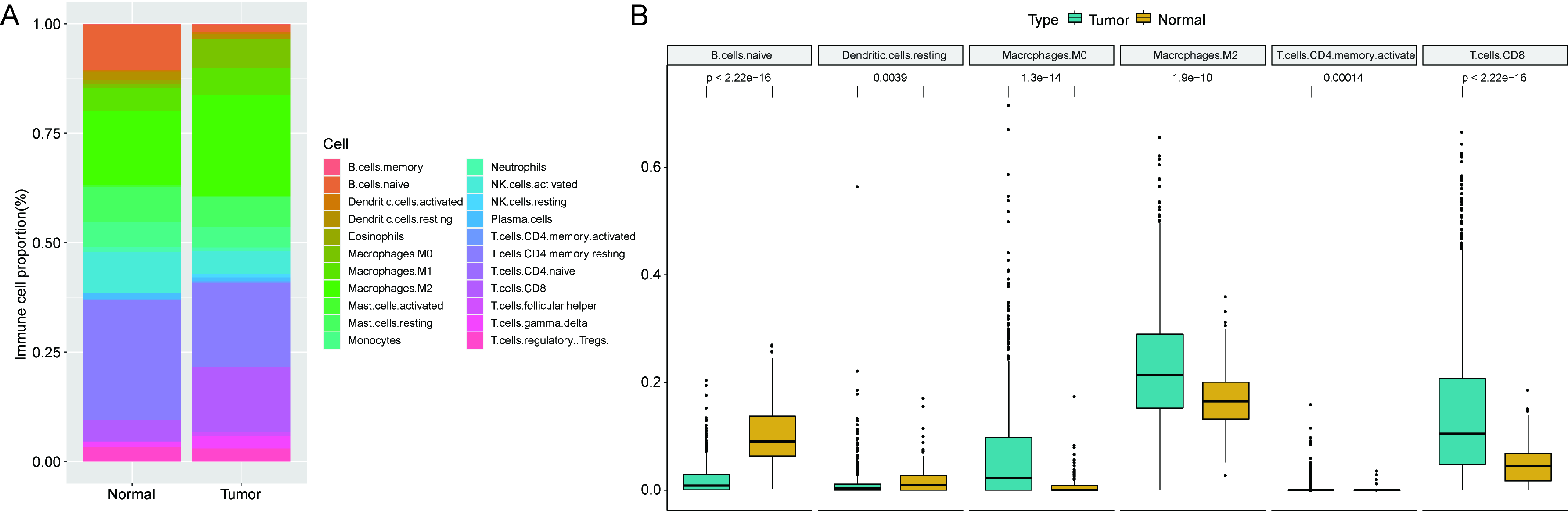

Supplement: Supplementary file 2 — Additional file 2 Fig. S1. Relative proportions of immune cells in TCGA dataset analyzed by CIBERSORT. a The colum plot of 22 types of immune cells in TCGA renal cancer and paracancerous samples. b The box plot of 6 types of immune cells with significant differences between renal cancer and paracancerous samples. [file 12885_2021_8244_MOESM2_ESM.tif]

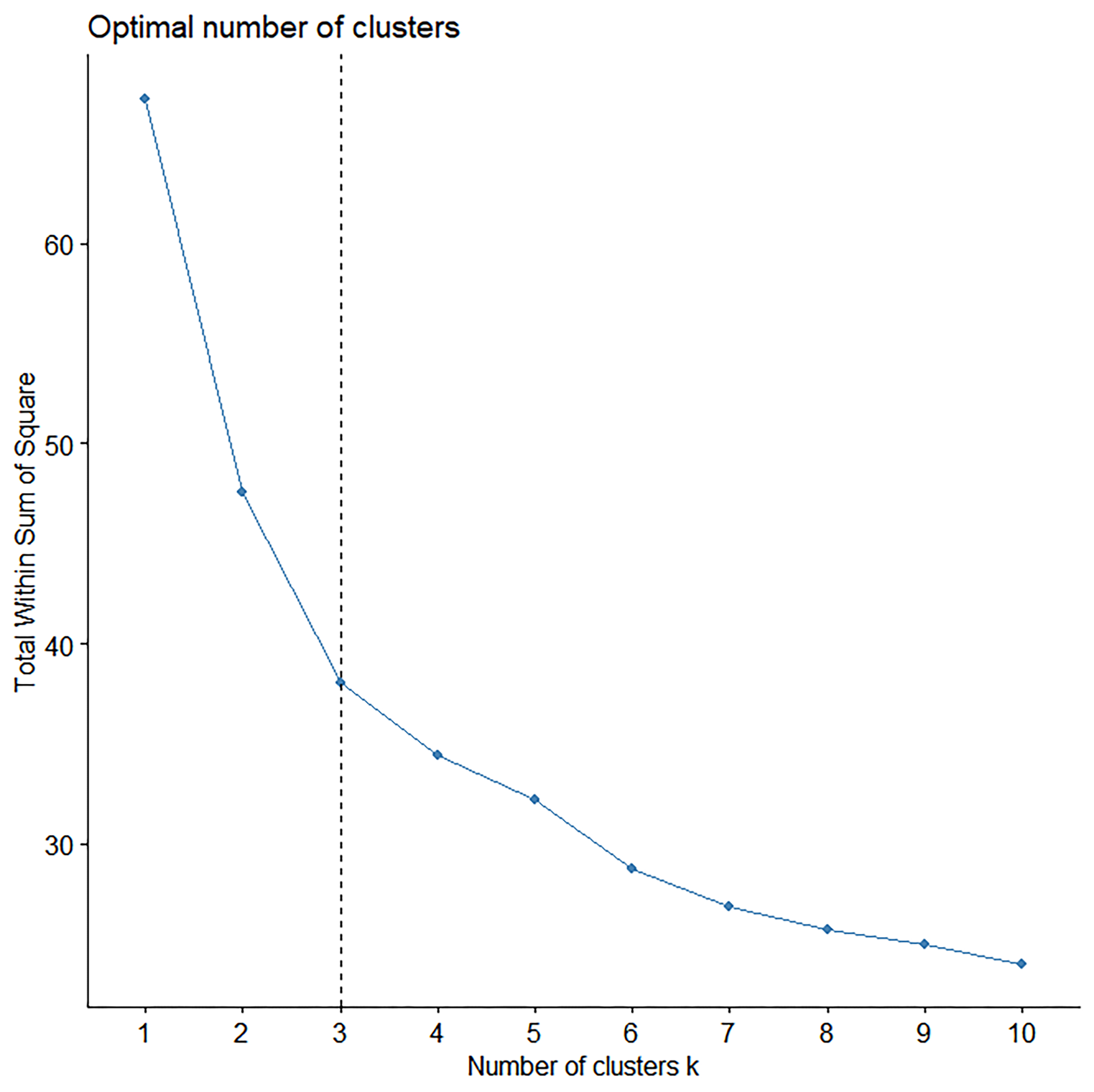

Supplement: Supplementary file 3 — Additional file 3 Fig. S2. Selection of the optimal number of clusters. [file 12885_2021_8244_MOESM3_ESM.tif]

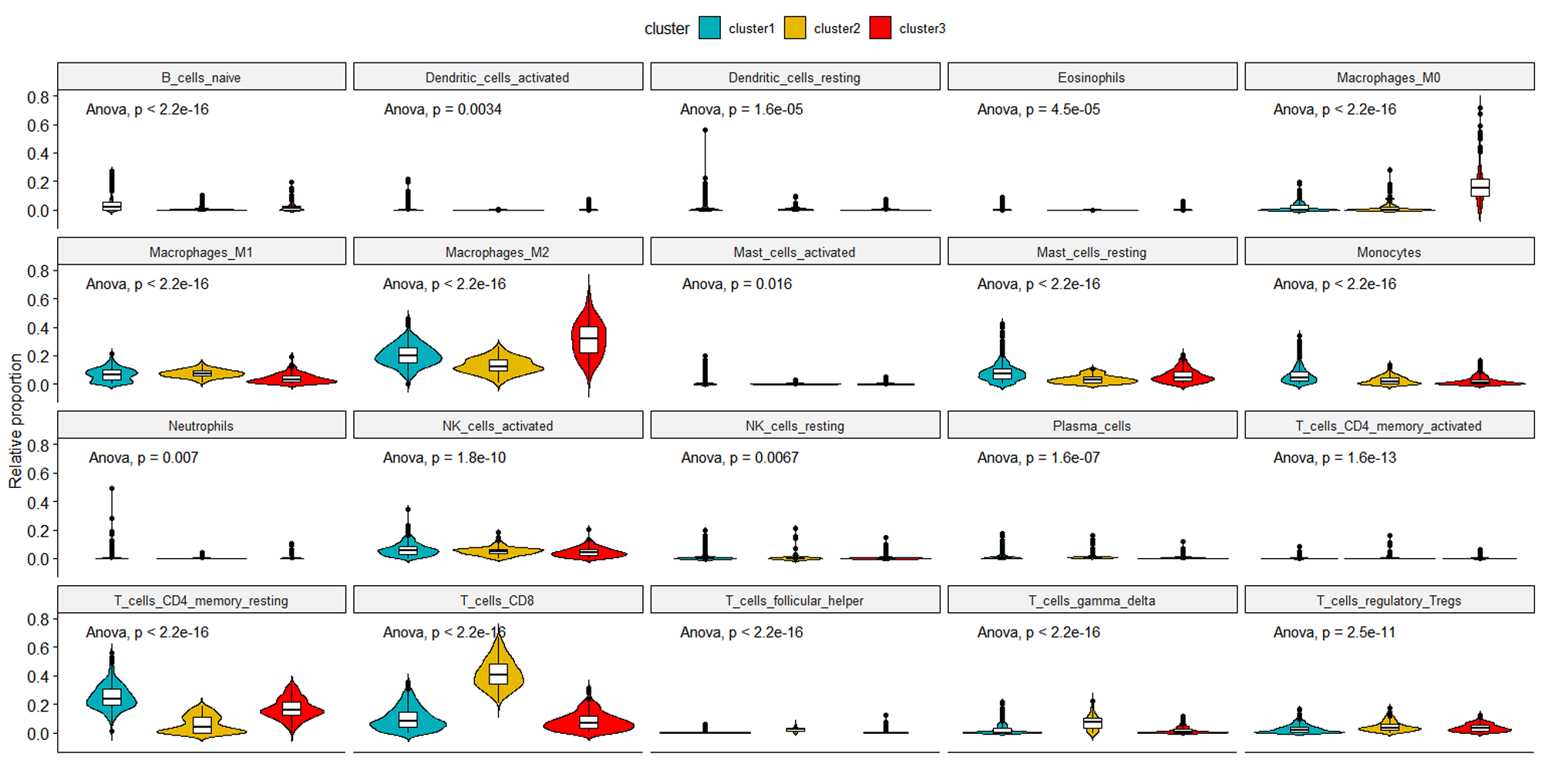

Supplement: Supplementary file 4 — Additional file 4 Fig. S3. The violin plot of immune cell type abundances differ between clusters. Violin colors represent different clusters, and the vertical axis represents the relative proportion of immune cells. [file 12885_2021_8244_MOESM4_ESM.tif]

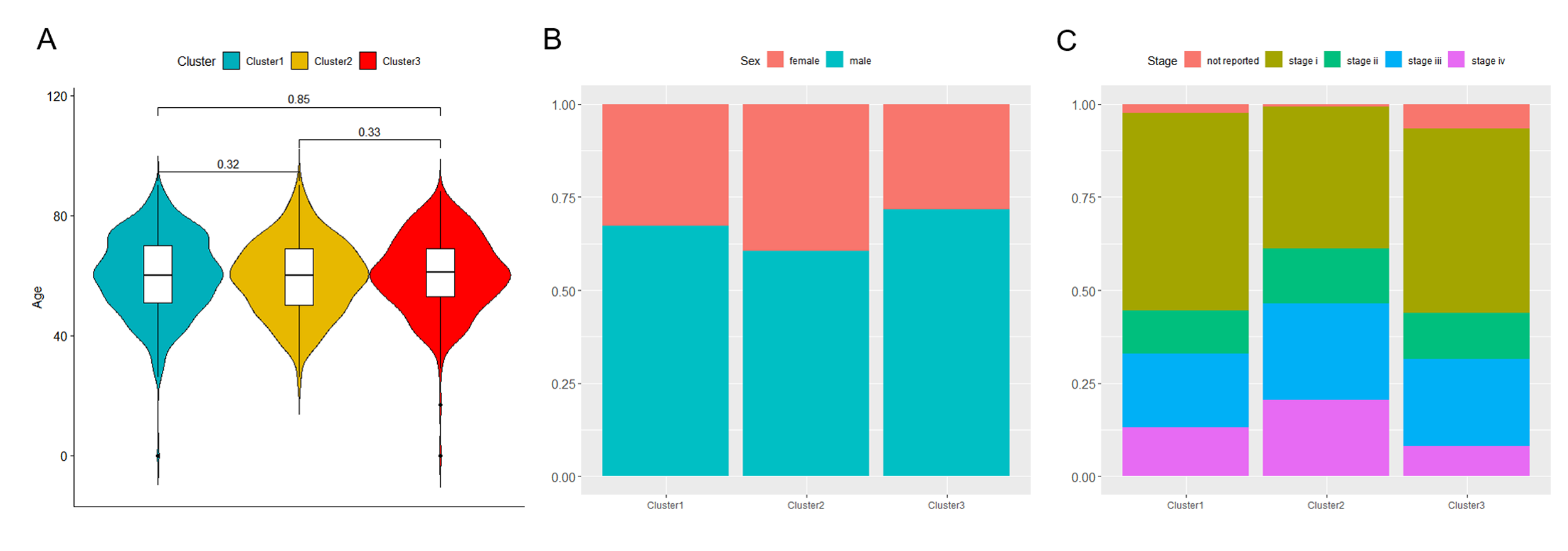

Supplement: Supplementary file 5 — Additional file 5 Fig. S4. The violin plot of clinical characters in three clusters. Violin colors represent different clusters, and the vertical axis represents samples. a Age. b Sex. c Stage. [file 12885_2021_8244_MOESM5_ESM.tif]
